# Land cover, land use changes and air pollution in Asia: a synthesis

**DOI:** 10.1088/1748-9326/aa9c5d

**Published:** 2017-12-14

**Authors:** Krishna Vadrevu, Toshimasa Ohara, Chris Justice

**Affiliations:** 1 NASA Marshall Space Flight Center, Huntsville, AL, United States of America; 2 National Institute of Environmental Studies, Tsukuba, Japan; 3 University of Maryland, College Park, MD, United States of America

**Keywords:** land cover and land use changes, population, air pollution, interdisciplinary approach, south and southeast Asia, synthesis

## Abstract

A better understanding of land cover/land use changes (LCLUC) and their interactions with the atmospheric environment is essential for the sustainable management of natural resources, environmental protection, air quality, agricultural planning and food security. The 15 papers published in this focus issue showcase a variety of studies relating to drivers and impacts of LCLUC and air pollution in different South/Southeast Asian (S/SEA) countries. This synthesis article, in addition to giving context to the articles in this focus issue, also reviews the broad linkages between population, LCLUC and air pollution. Additionally, we identify knowledge gaps and research priorities that are essential in addressing air pollution issues in the region. We conclude that for effective pollution mitigation in S/SEA countries, quantifying drivers, sources and impacts of pollution need a thorough data analysis through ground-based instrumentation, models and integrated research approaches. We also stress the need for the development of sustainable technologies and strengthening the scientific and resource management communities through capacity building and training activities to address air pollution issues in S/SEA countries.

## 1. Introduction

**Figure 1:**
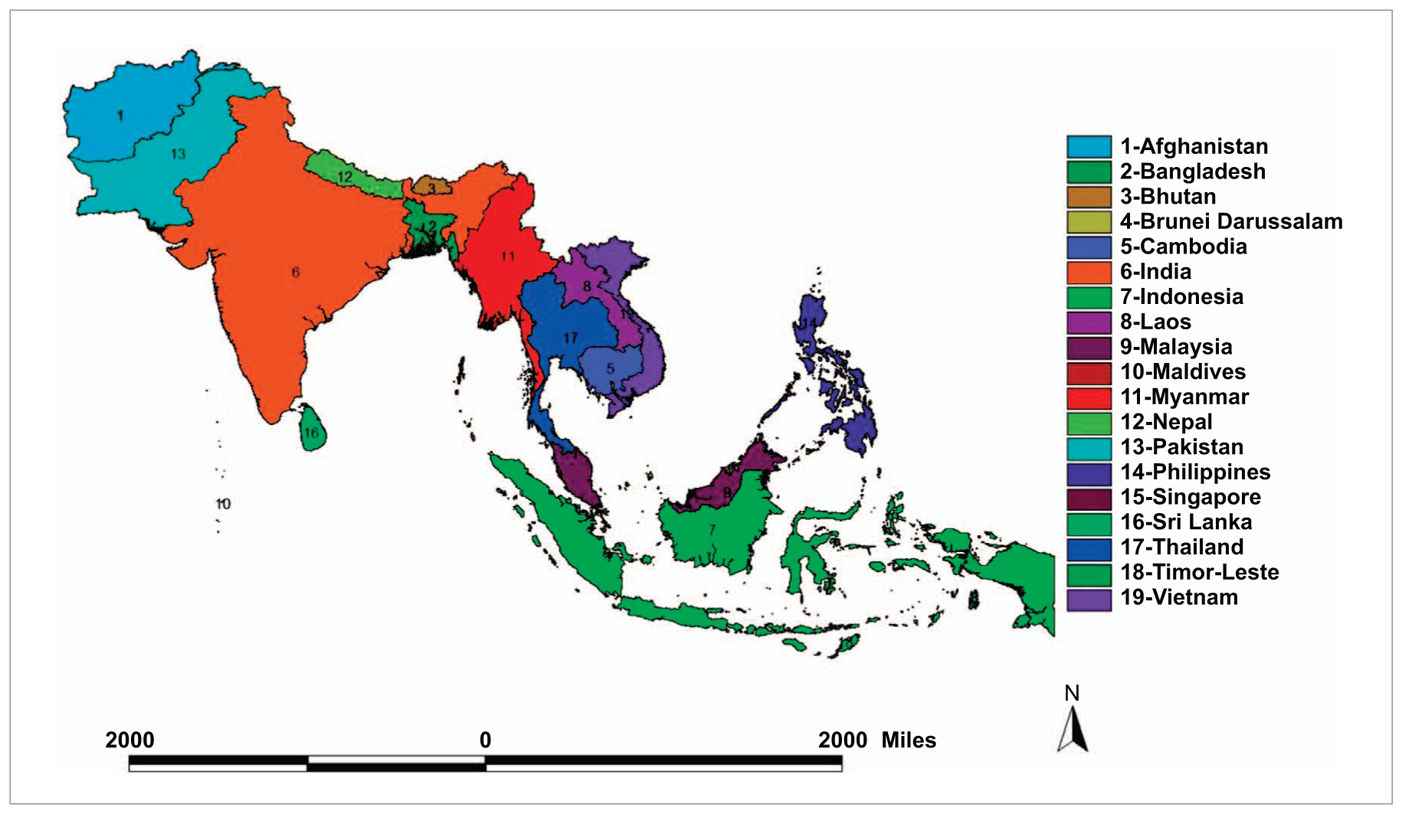
South/Southeast Asian countries.

The countries of South and Southeast Asia (S/SEA) (figure 1) span an area of about 9.75 million km^2^ and have a population of 2.36 billion. They represent almost 30.66% of the world’s population in only 6.57% of the world’s land area. Population growth and urbanization have caused immense pressure to convert land from natural and agricultural areas into residential and urban uses with significant impacts on ecosystem services (Justice *et al* 2015). Several major cities in S/SEA have air quality issues, most of which can be attributed to rapid industrialization, urbanization and increased demand from the energy sector (Foell *et al* 1995, Ohara and Murano 2001, Gurjar *et al* 2016). Addressing these land cover/land use changes (LCLUC) issues and associated air pollution is an important topic addressed by the South/Southeast Asia Research Initiative (SARI) which is funded by the NASA LCLUC program (www.lcluc.umd.edu). The goal of SARI is to develop an innovative regional research, education, and capacity building program involving state-of-the-art remote sensing, engineering, natural and social sciences to enrich LCLUC science in S/SEA. The SARI objectives are twofold; to advance LCLUC science in the region, and to strengthen existing, and build new, collaborations between US and S/SEA researchers in the areas of LCLUC research. To address LCLUC science, SARI has been utilizing a systems approach to problem-solving that examines both biophysical and socioeconomic aspects of land systems, including the interactions between land use and climate and the interrelationships among policy, governance, and land use. A central component of SARI is to use geospatial data from both remotely sensed and *in situ* sources and models to further LCLUC research. In this article, we synthesize the linkages between population trends, LCLUC and greenhouse gas emissions (GHGs) in S/SEA countries (figure 1) (hereafter mentioned as SARI countries throughout the article). We also highlight the important research needs and priorities for LCLUC and air pollution research in the following sections through synoptic analysis of data from the United Nations Food and Agriculture Organization (FAOSTAT 2017) and papers published in this focus issue.

## 2. Population and energy consumption are highly related in S/SEA—thus efforts should focus on managing population growth rates

**Figure 2:**
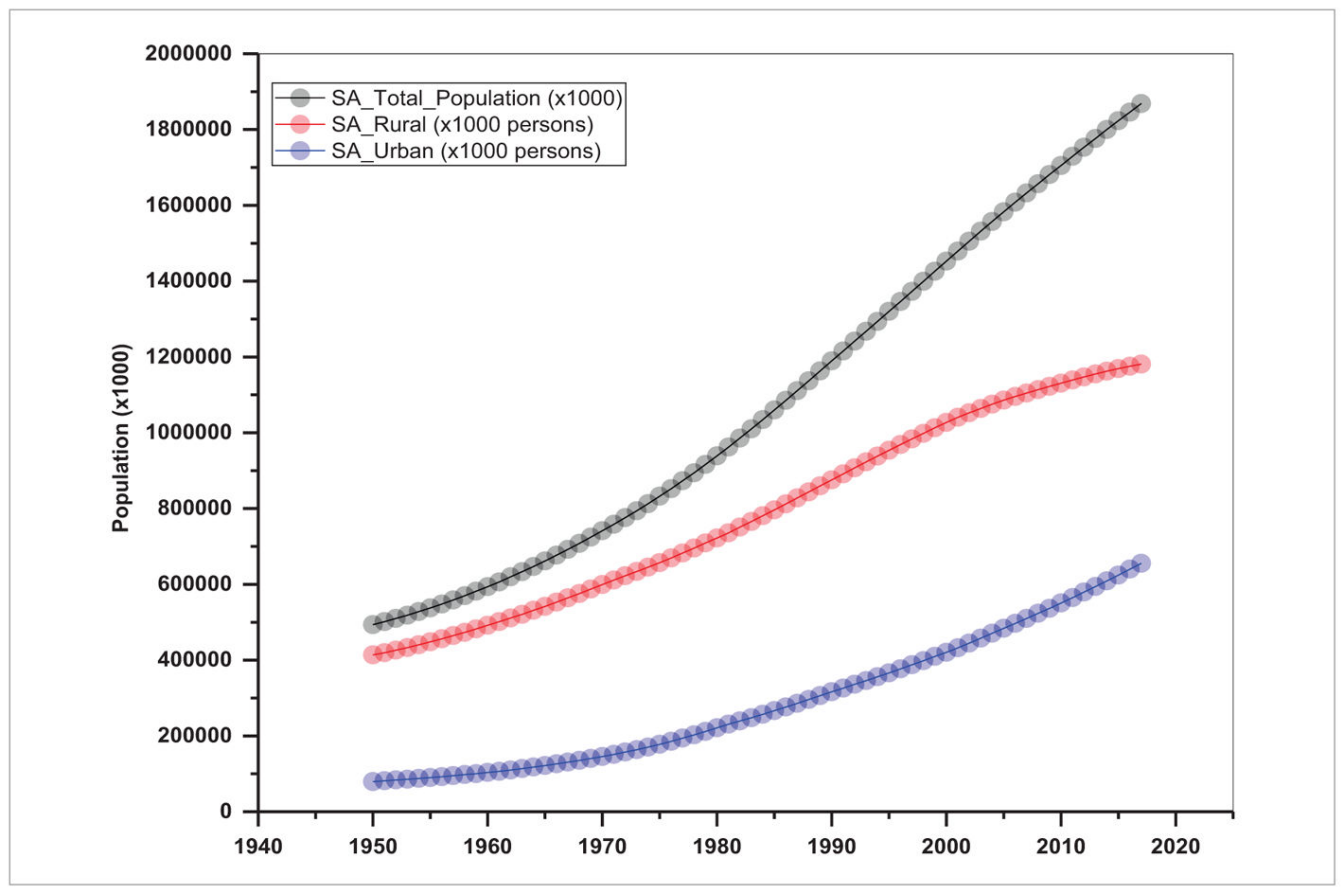
Trends in population in South Asia.

**Figure 3:**
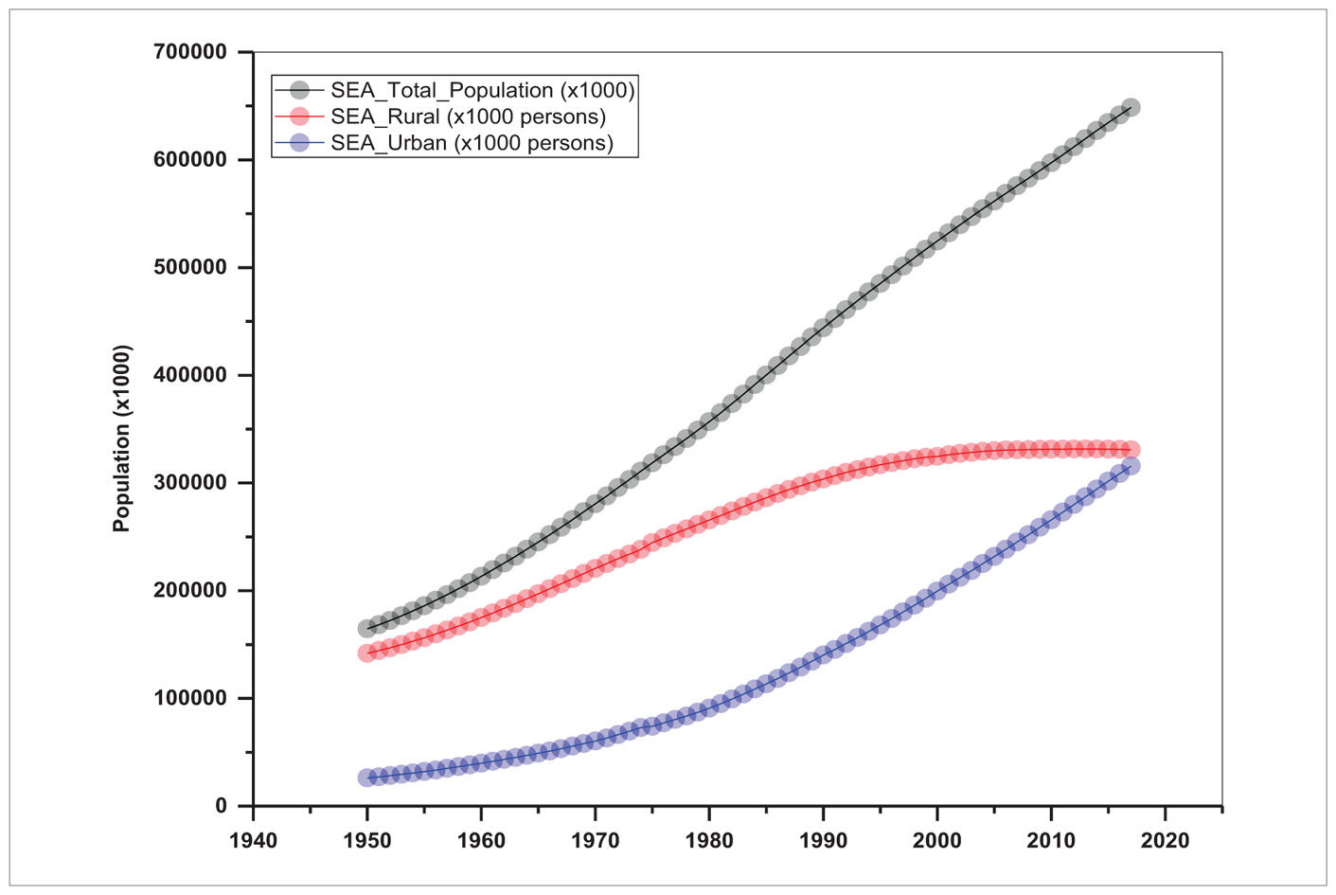
Trends in population in Southeast Asia.

**Figure 4:**
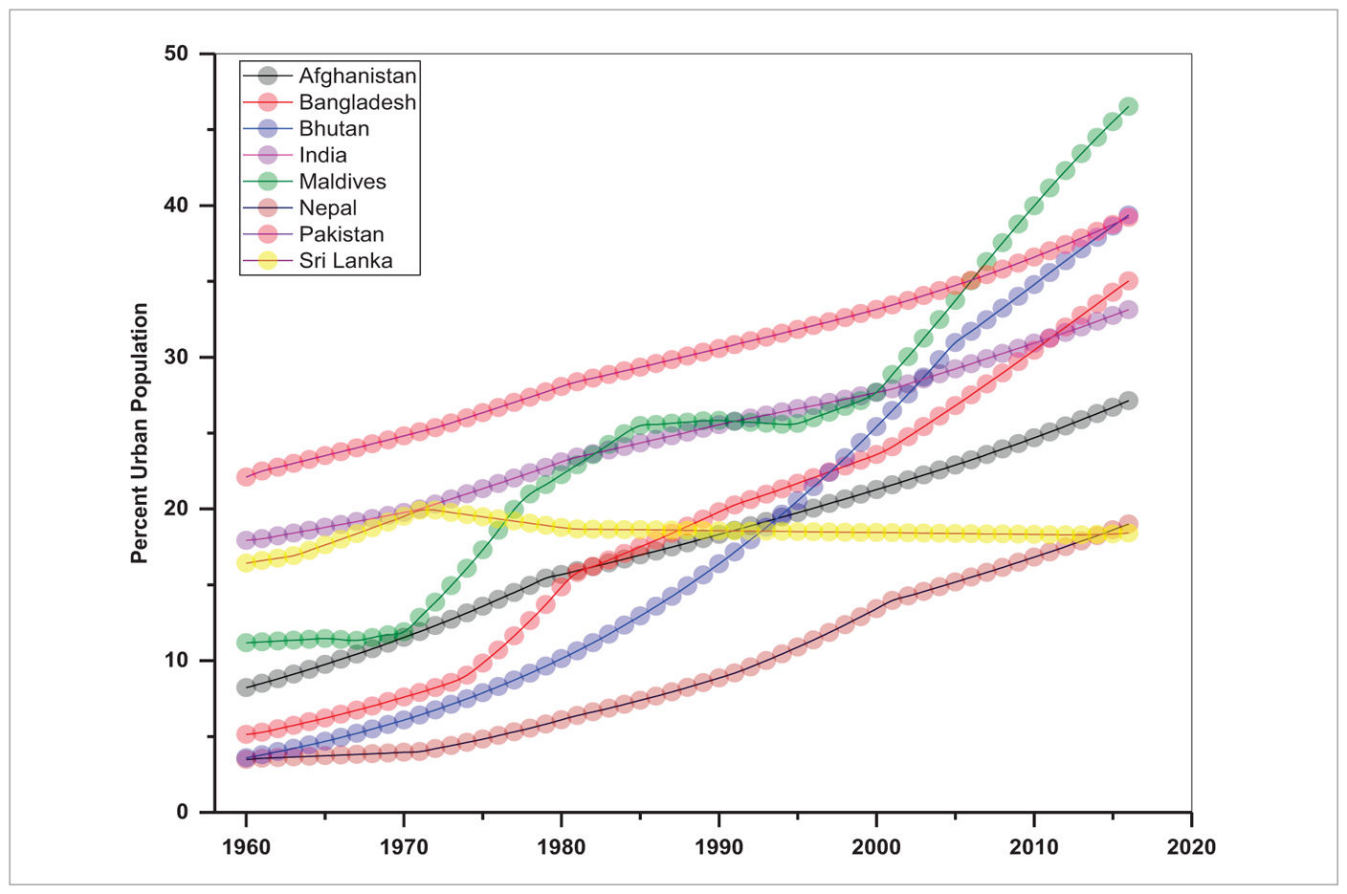
Urban population (% of total) in different South Asian countries.

**Figure 5:**
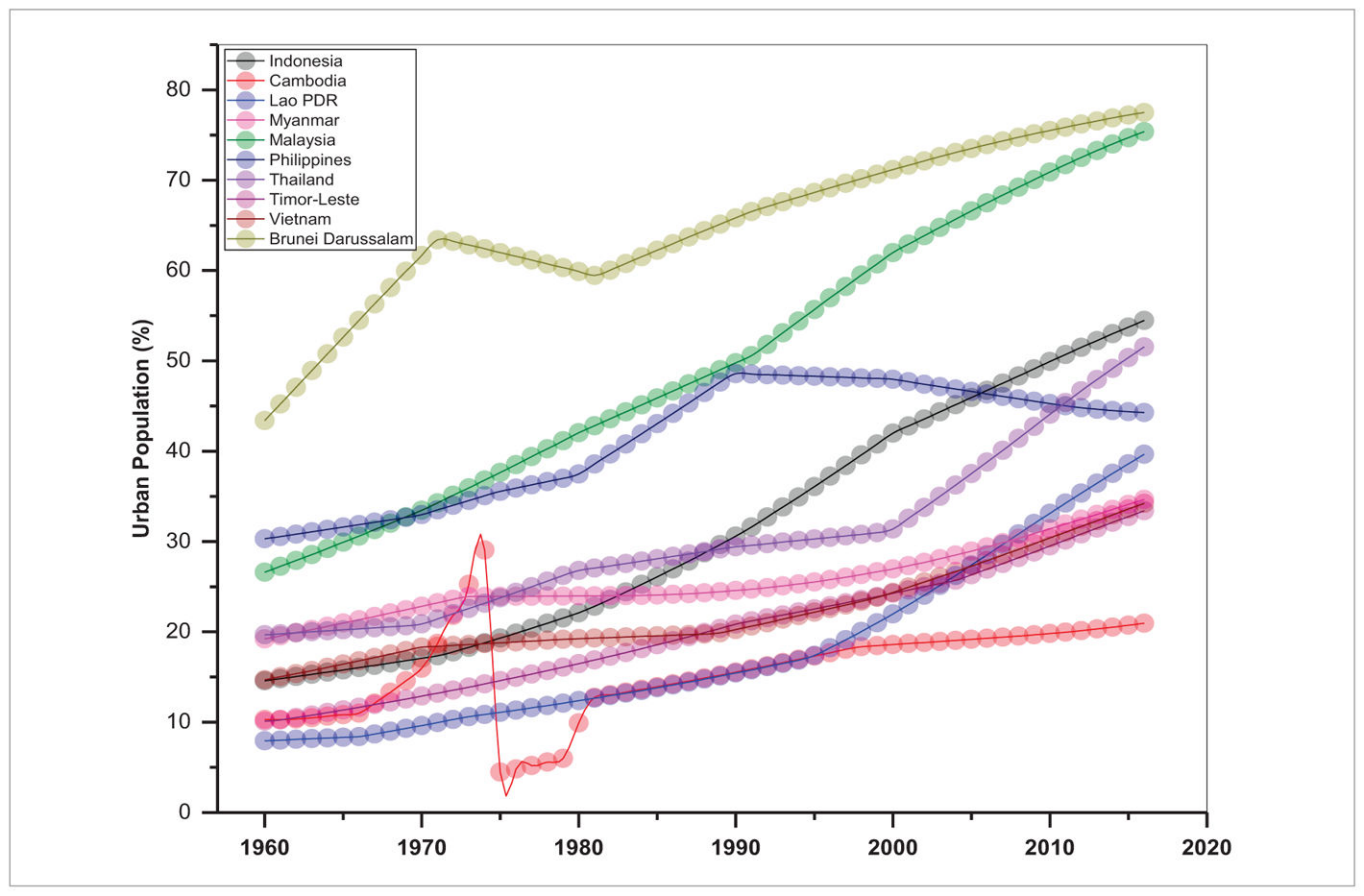
Urban population (% of total) in different Southeast Asian countries.

In several SARI countries, urban dwellings have fueled increasing energy demand from automobiles, domestic heating, and small-scale industries (Krey *et al* 2012, Kammen and Sunter 2016). Trends in total population including rural and urban population aggregated for S/SEA countries are given in figures 2 and 3 and the urban population percent for individual countries in figures 4 and 5. These figures suggest a relatively high population, nearly 1.8 billion for South Asia compared to 0.63 billion in Southeast Asia. Also, in South Asia, there is still a larger rural population than an urban population (figure 2), whereas in Southeast Asia, the rural population is stagnating while the urban population is expanding (figure 3). Also, the trends suggest a significant increase in the percentage of the population who live in urban areas from 11.0% in 1960 to 32% in 2016 in South Asian countries and 19.68%–46.60% for the same time period in Southeast Asian countries (excluding Singapore, as the country has been 100% urban since 1960). Trends in urban population percentage from 1960 until recently for S/SEA countries are given in figures 4 and 5. These trends suggest that in South Asia, the Maldives, Bangladesh and Bhutan had the highest urban population increase whereas in Nepal and Sri Lanka, the urban growth was the lowest. Similarly, in Southeast Asia, Brunei Darussalam, Malaysia and Indonesia had the highest urban population percent growth whereas regionally, Cambodia, Timor-Leste and Vietnam had the least urban population growth.

**Figure 6:**
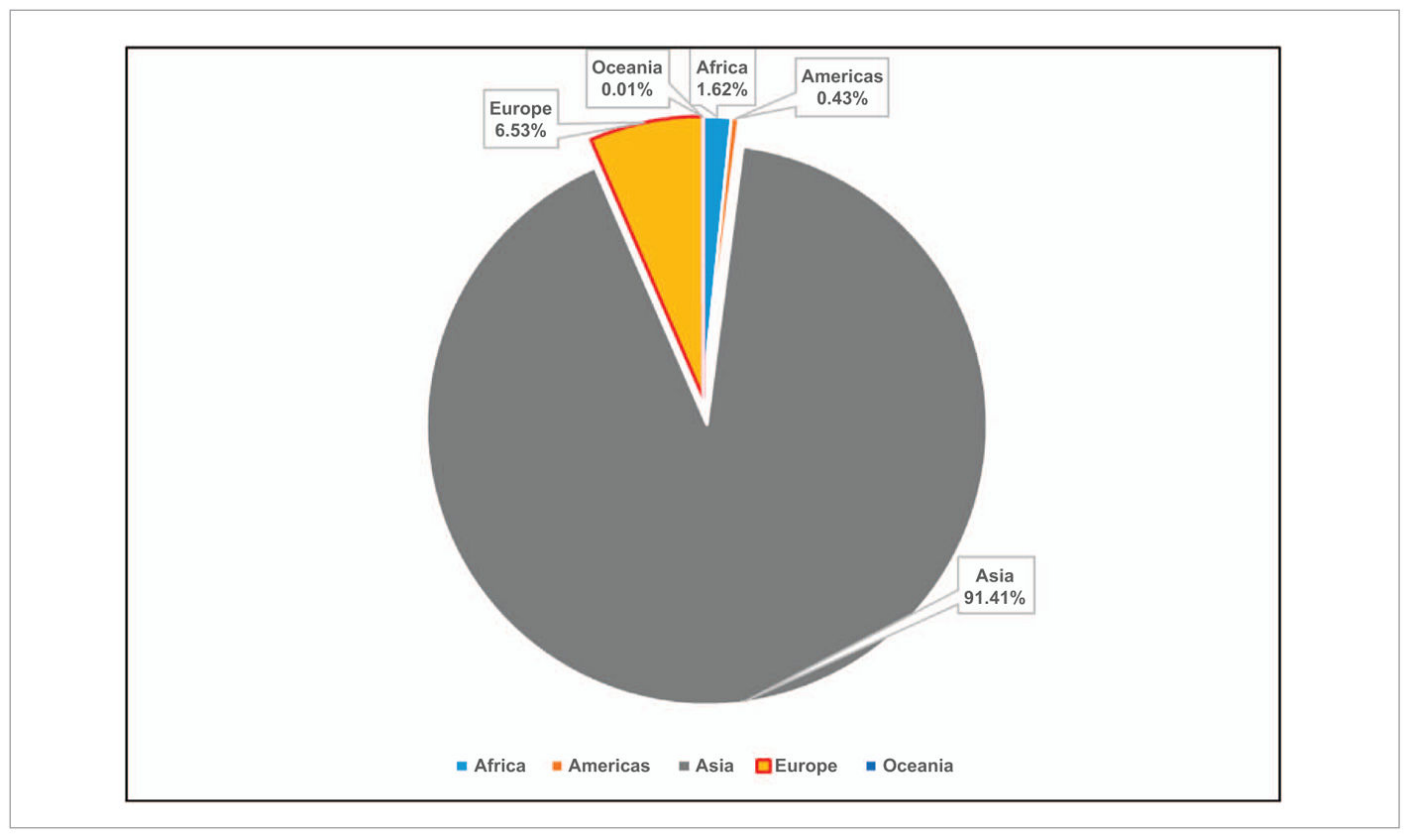
Averaged (1990–2012) CO_2_ emissions (%) from coal by continent.

**Figure 7:**
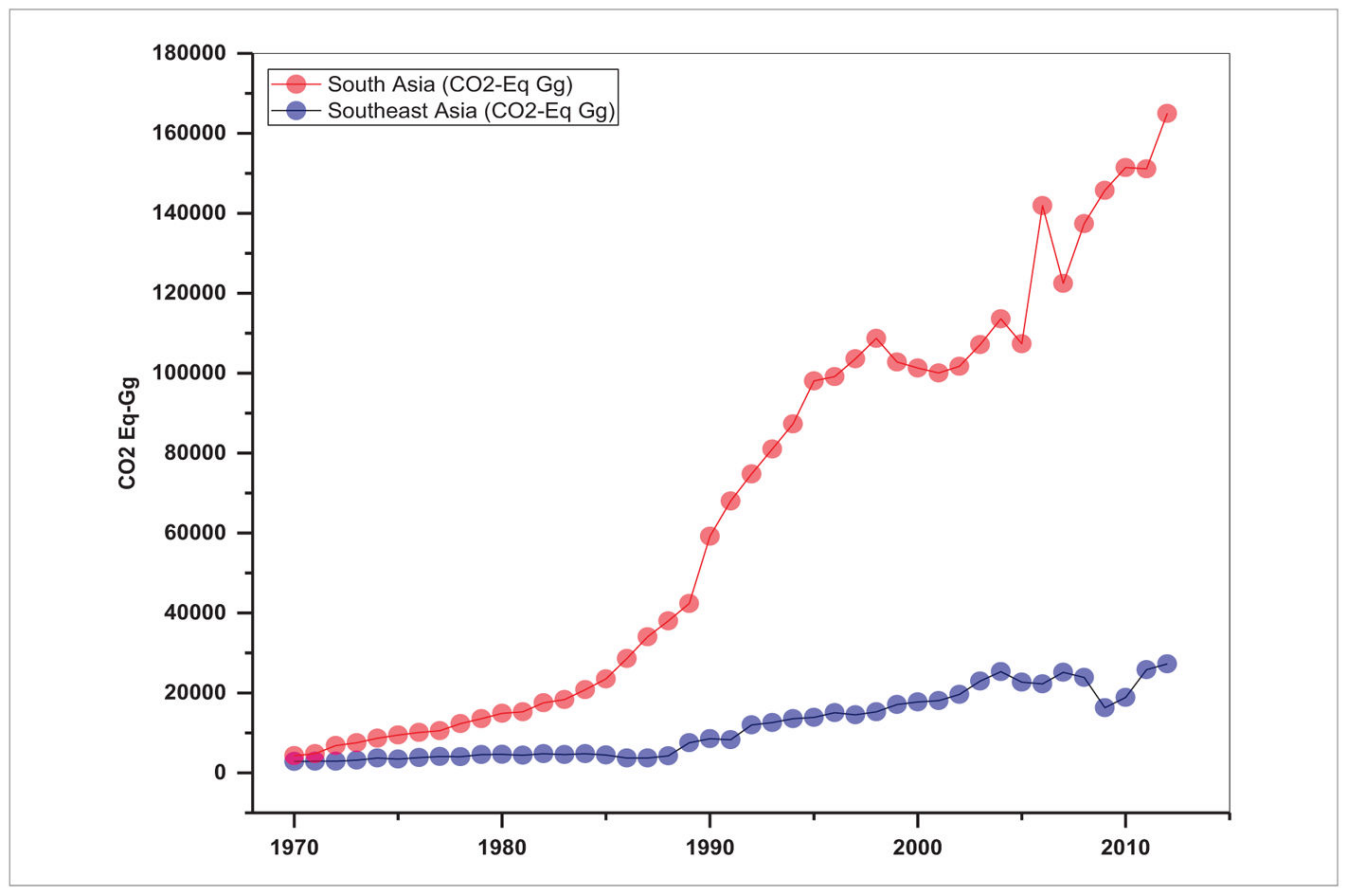
Trends in energy use in South and Southeast Asian regions (1970–2012).

**Figure 8:**
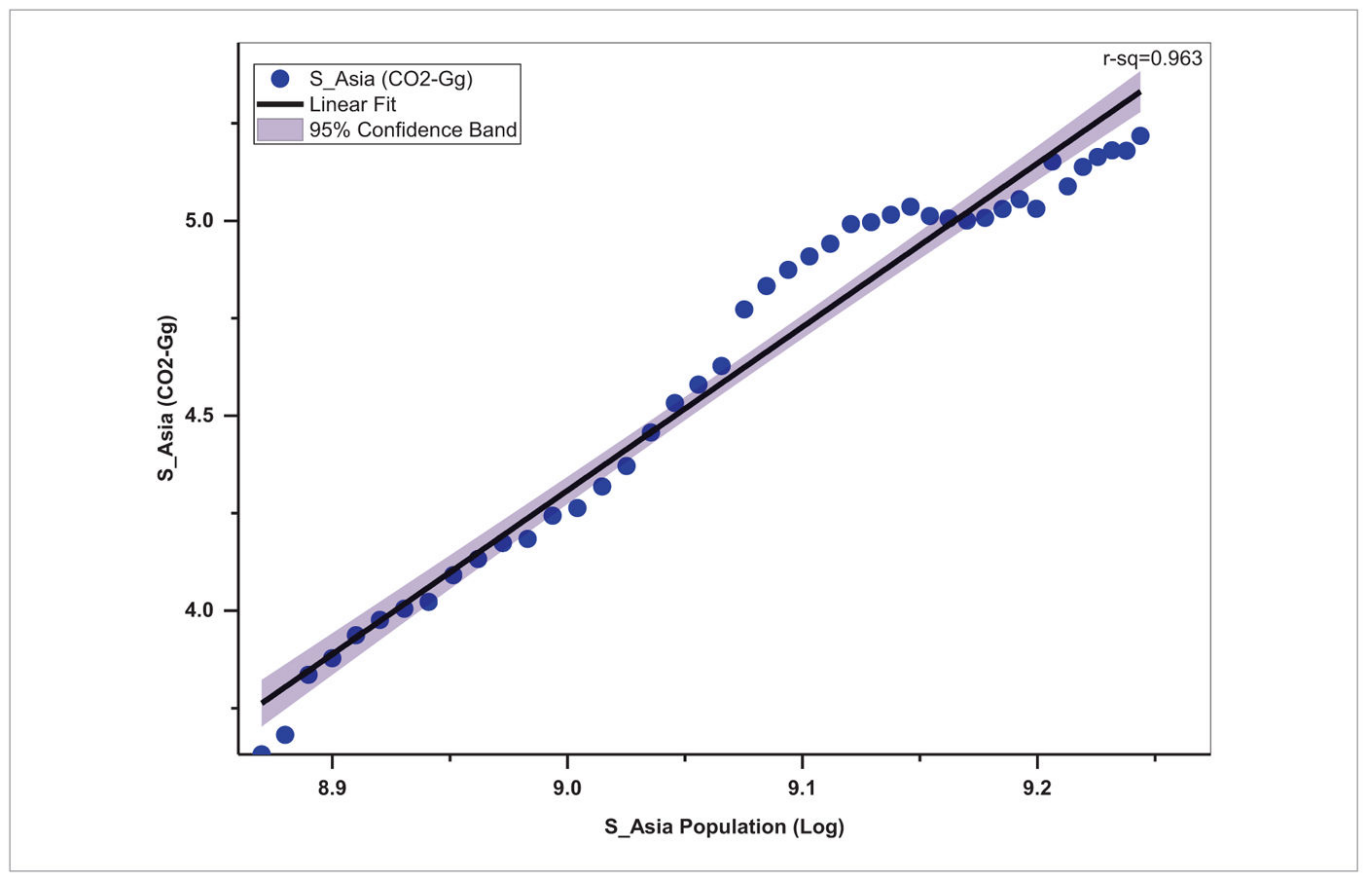
Population and energy use in South Asia.

**Figure 9:**
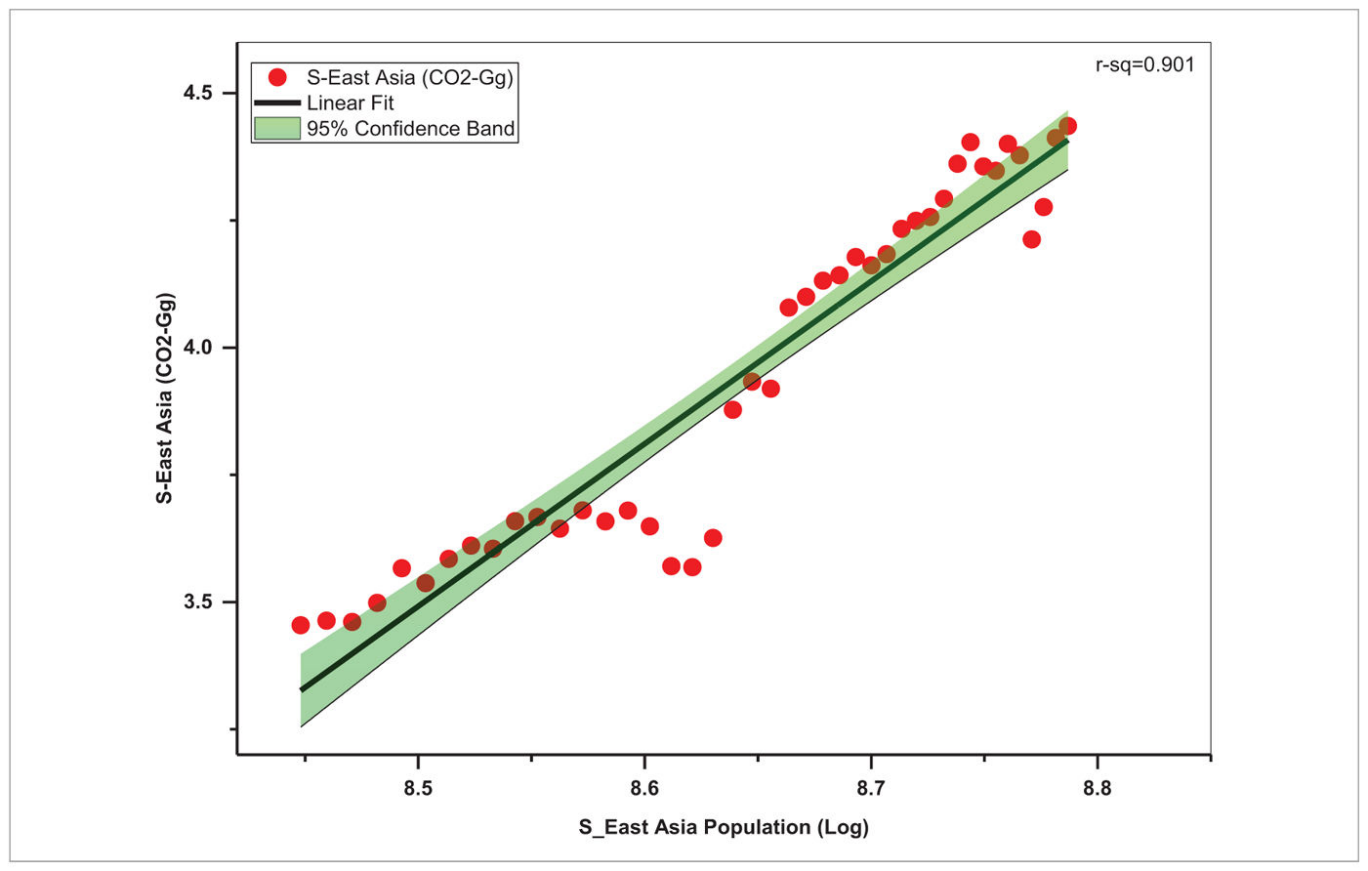
Population and energy use in Southeast Asia.

With coal as the cheapest and most widely accessible fuel, it is still the primary source of energy in Asia (Sovacool 2007, Lane 2017) accounting for about 91% of the total energy use and resulting CO_2_ emissions (figure 6). For example, Asia accounts for 90 837 Gg of CO_2_ equivalent emissions from coal, as compared to 429.35 Gg in the Americas, 1608.24 Gg in Africa, 6486.88 Gg in Europe and 11.41 Gg in Oceania (FAOSTAT 2017). Coal combustion also contributes significantly to sulfur and particulate matter pollution that often exceeds the World Health Organization (WHO) air quality guidelines in Asia, impacting human health (Gustafsson *et al* 2009, Koplitz *et al* 2017). Trends in energy use in terms of CO_2_-equivalent emissions in Gg (figure 7) suggest that in South Asia, the energy use increased from 4278.70 Gg in 1970 to 165 024 Gg during 2012, i.e. an annual growth rate of 89.44%. In contrast, in Southeast Asia, the energy use increased from 2846.41 Gg during 1970 to 27 257 Gg during 2012 with an annual growth rate of 20.41%. Population increase was relatively high with 3.25% annual growth in South Asian countries as a whole compared to 2.81% annual growth in Southeast Asian countries. Our analysis also suggests that energy related emissions (in terms of CO_2_-equivalent) are strongly dependent on total population, with a stronger correlation in South Asia (*r*^2^ = 0.96) (figure 8) than Southeast Asia (*r*^2^ = 0.90) (figure 9). Thus, managing population growth in the coming years will help in reducing energy related emissions in Asia.

## 3. Understanding drivers and impacts of LCLUC in S/SEA requires interdisciplinary approaches

**Figure 10:**
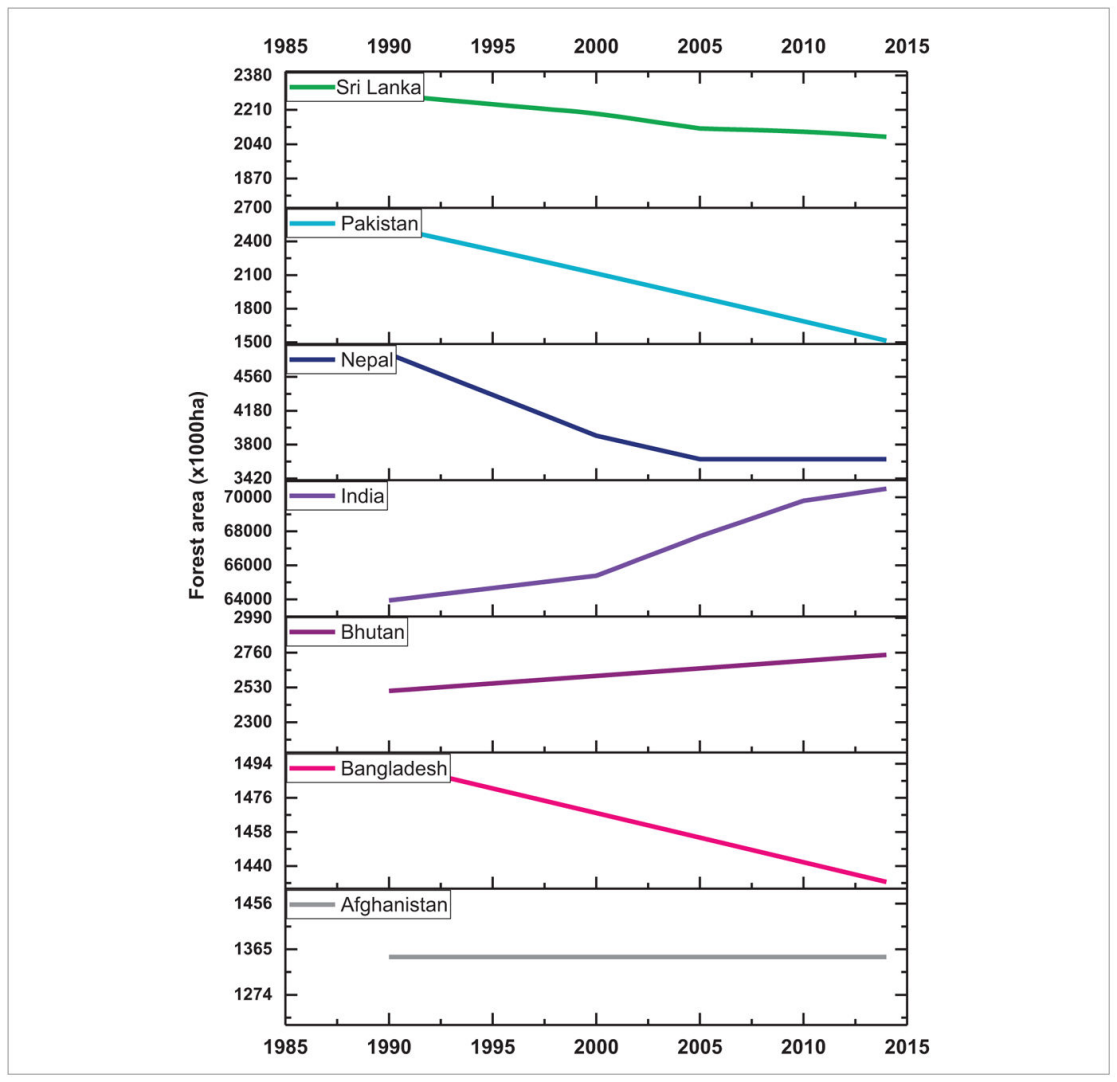
Trends in forest area in South Asian countries.

**Figure 11:**
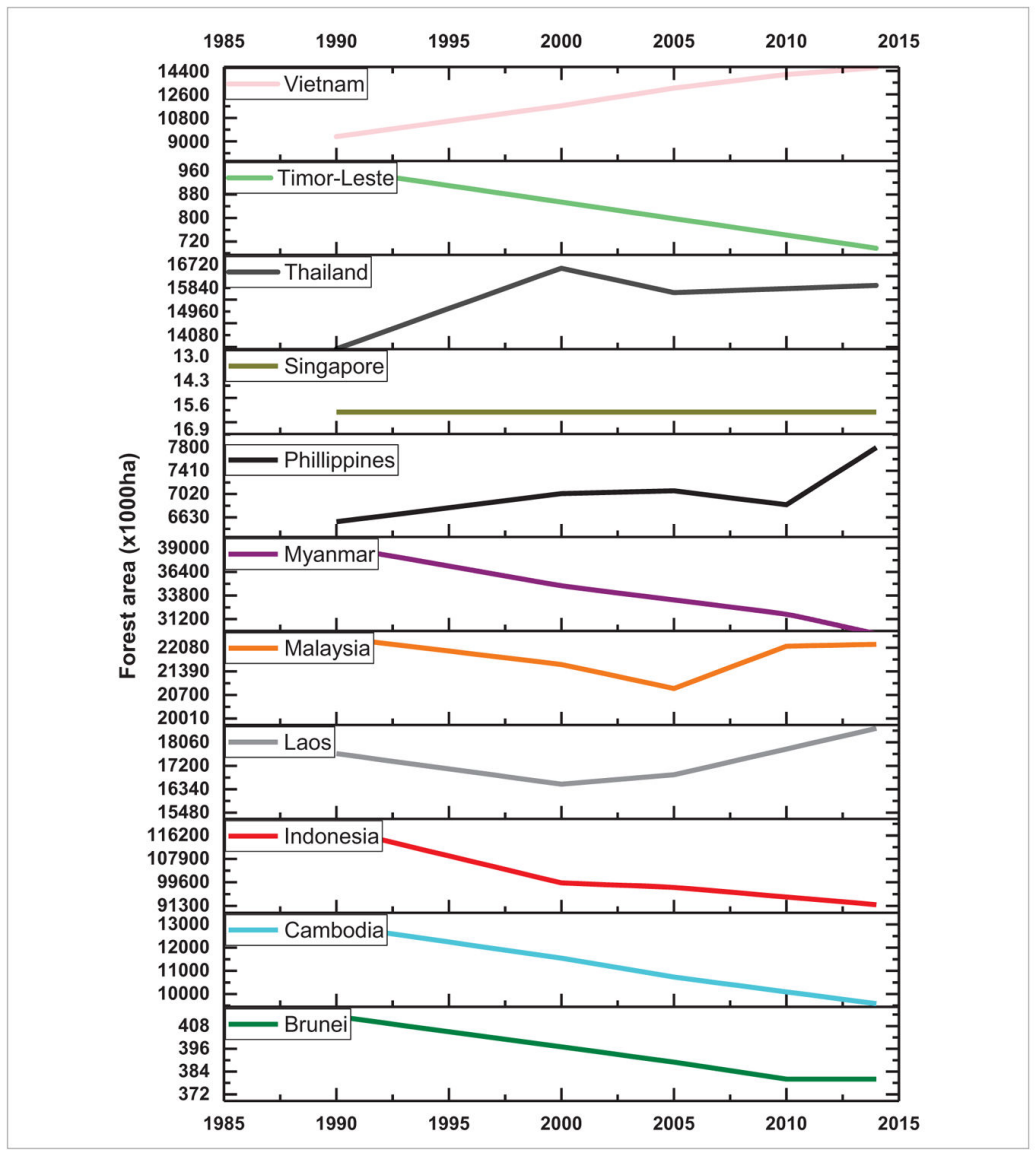
Trends in forest area in Southeast Asian countries.

Population pressure together with rapid economic development in SARI countries is causing immense pressure to convert land from forest to agriculture and from agricultural areas to residential and urban uses with significant impact on ecosystem services. Increased LCLUC in the region is impacting forest resources, biodiversity, regional climate, biogeochemical cycles, water resources and other ecosystem services (Justice *et al* 2015). Mostly, LCLUC in SARI countries is powered by increased food demand for their growing population. Several countries are transitioning from largely agrarian to urban societies due to increased industrialization. Forests provide a natural environment and have an increasing human footprint through deforestation/afforestation which is clearly evident in SARI countries (figures 10 and 11). Trends in forest area from 1985–2014 suggest that in Sri Lanka, Pakistan, Nepal and Bangladesh, forest area decreased considerably whereas it is reported to have increased in India and Bhutan (FAOSTAT 2017, figure 10). In Southeast Asia, Timor-Leste, Myanmar, Indonesia, Cambodia and Brunei, forest area decreased from 1985–2014 whereas it increased in Vietnam, Thailand and Philippines (figure 11). These changes cannot be directly linked to population growth alone; for example, although population increased significantly in India, the forest cover did not decline—in contrast, it increased (figure 10). Although several direct causes such as agricultural expansion, overgrazing, timber extraction, demand for fuelwood, urban expansion and conversion to monoculture plantations, are well known, it is now widely recognized that multiple processes work simultaneously or sequentially to cause deforestation (Barbier and Burgess 2001).

**Figure 12:**
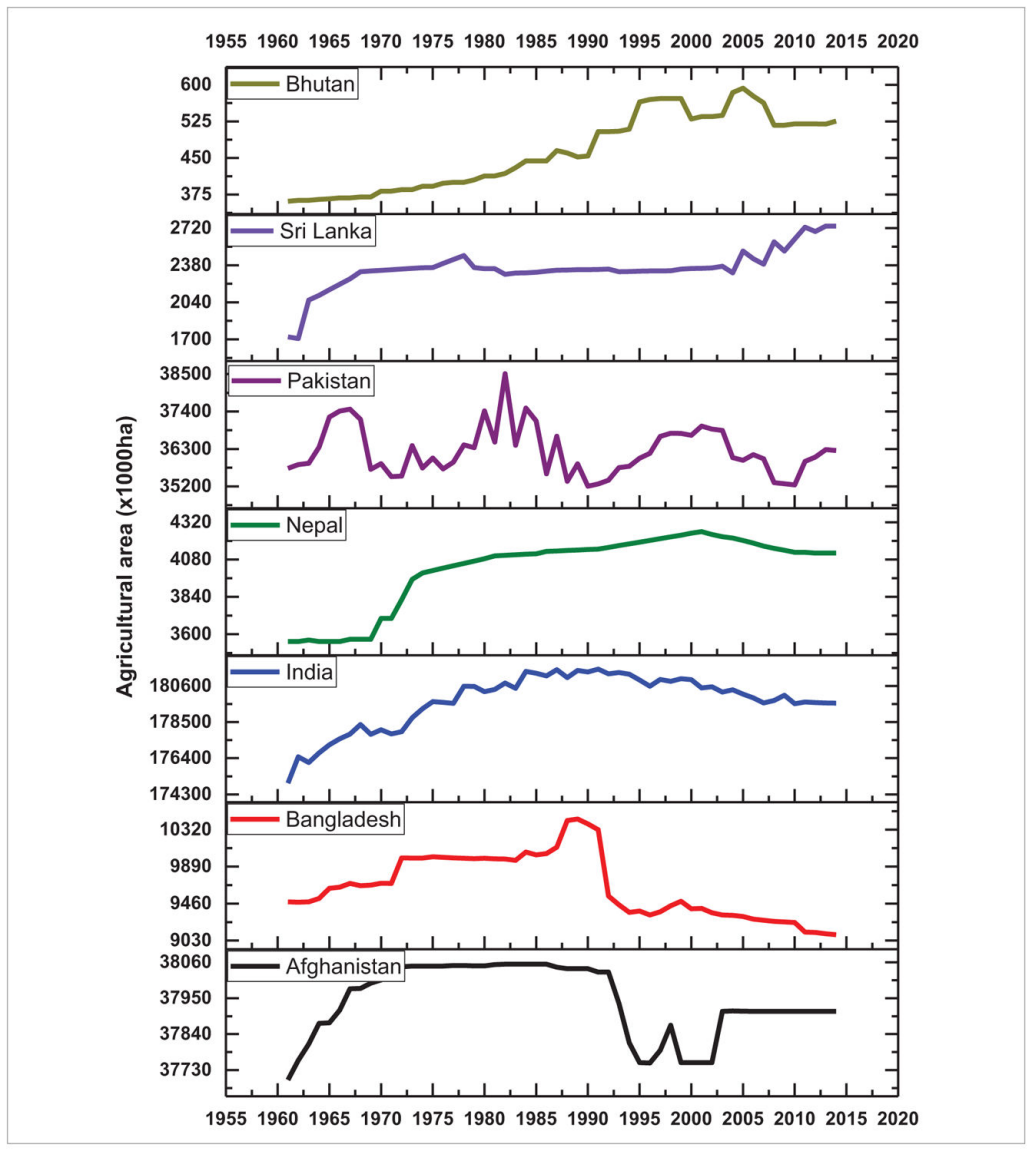
Trends in agricultural areas in South Asian countries.

**Figure 13:**
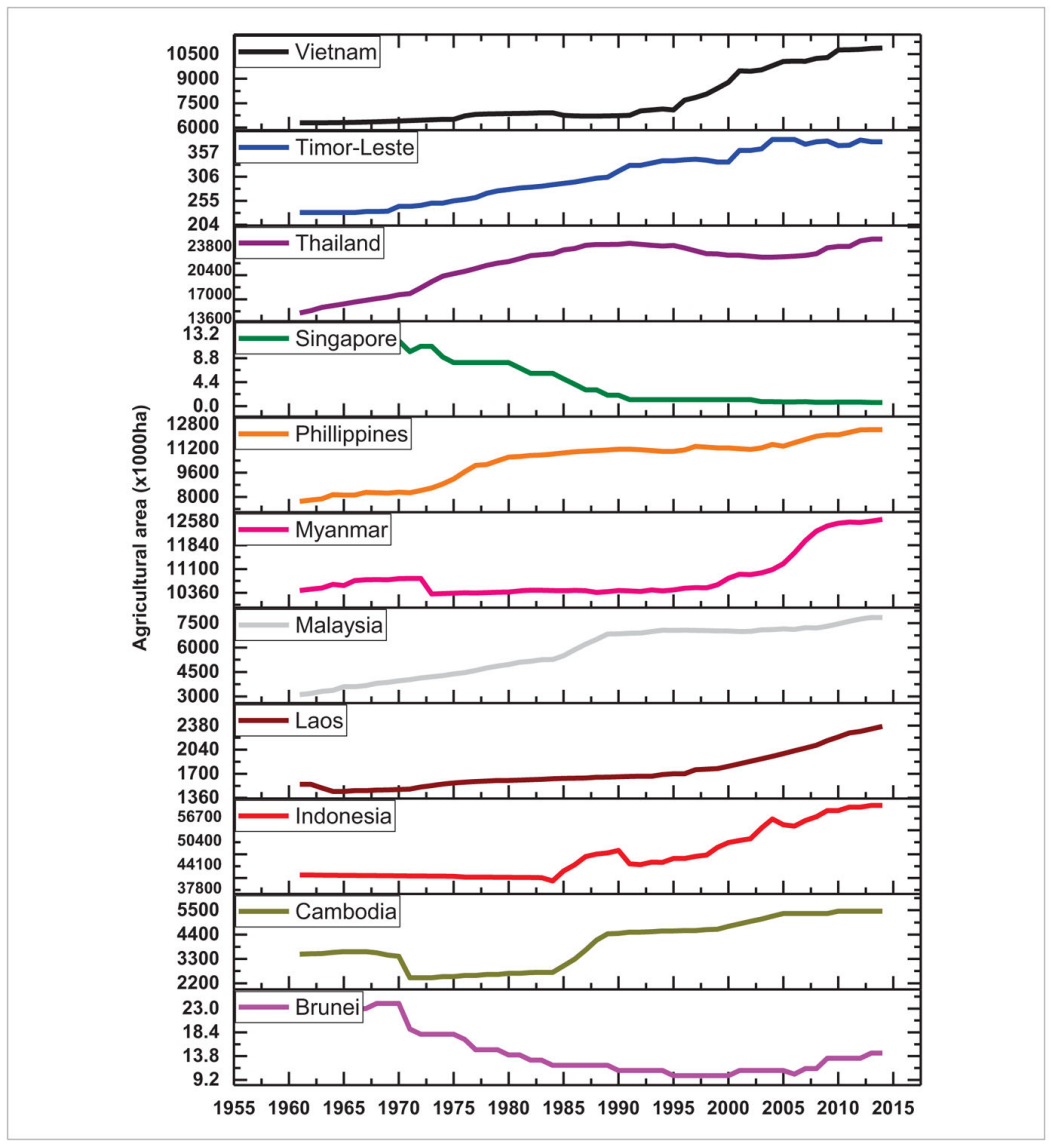
Trends in agricultural area in Southeast Asian countries.

Of the different causes of deforestation, it is well recognized that the single biggest direct cause is conversion to cropland and pasture, i.e. agricultural area expansion, mostly for subsistence needs (Geist and Lambin 2002, Kuusela and Amacher 2016). Trends in agricultural area expansion from 1960–2015 in SARI countries are shown in figures 12 and 13. The trends suggest that in almost all of the South Asian countries, agricultural areas expanded from 1960–2000 and then started to decline whereas in most of the Southeast Asian countries (Vietnam, Timor-Leste, Thailand, Philippines, Myanmar, Malaysia, Laos, Indonesia and Cambodia) agricultural expansion is still on-going (figure 12). Specifically, forest area loss in some countries such as Timor-Leste, Myanmar, Indonesia (Marlier *et al* 2015), and Cambodia can be directly linked to agricultural expansion. Recent studies also suggests that in addition to subsistence driven deforestation, expansion of large scale commercial plantations such as rubber and oil palm in Southeast Asian countries is driving deforestation (Wilcove and Koh 2010, Ziegler *et al* 2009, Vijay *et al* 2016). Economic development, agricultural subsidies, timber concessions, and global economic factors such as a country’s foreign debt, etc, can also drive deforestation (Carr *et al* 2005, Shandra *et al* 2016). Thus, quantifying the drivers of deforestation requires a more holistic approach integrating both biophysical as well as socioeconomic data in an interdisciplinary framework which is an important area of research in SARI countries. The inclusion of socioeconomics in land use research can yield fruitful results, which is well recognized in the LCLUC community, the NASA LCLUC program (www.lcluc.umd.edu) and SARI (www.sari.umd.edu).

**Figure 14:**
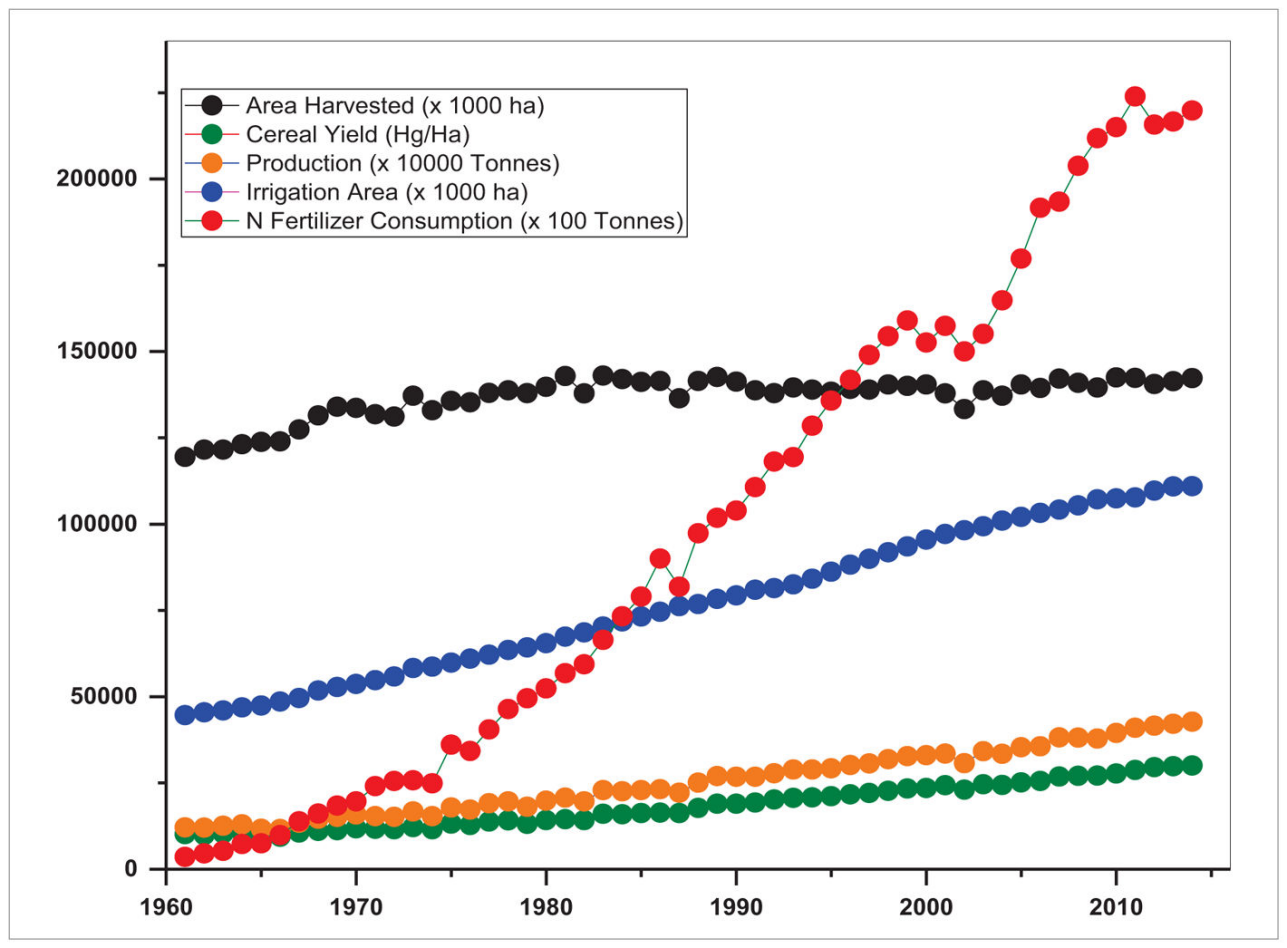
Trends in agricultural intensification indicators in South Asia.

**Figure 15:**
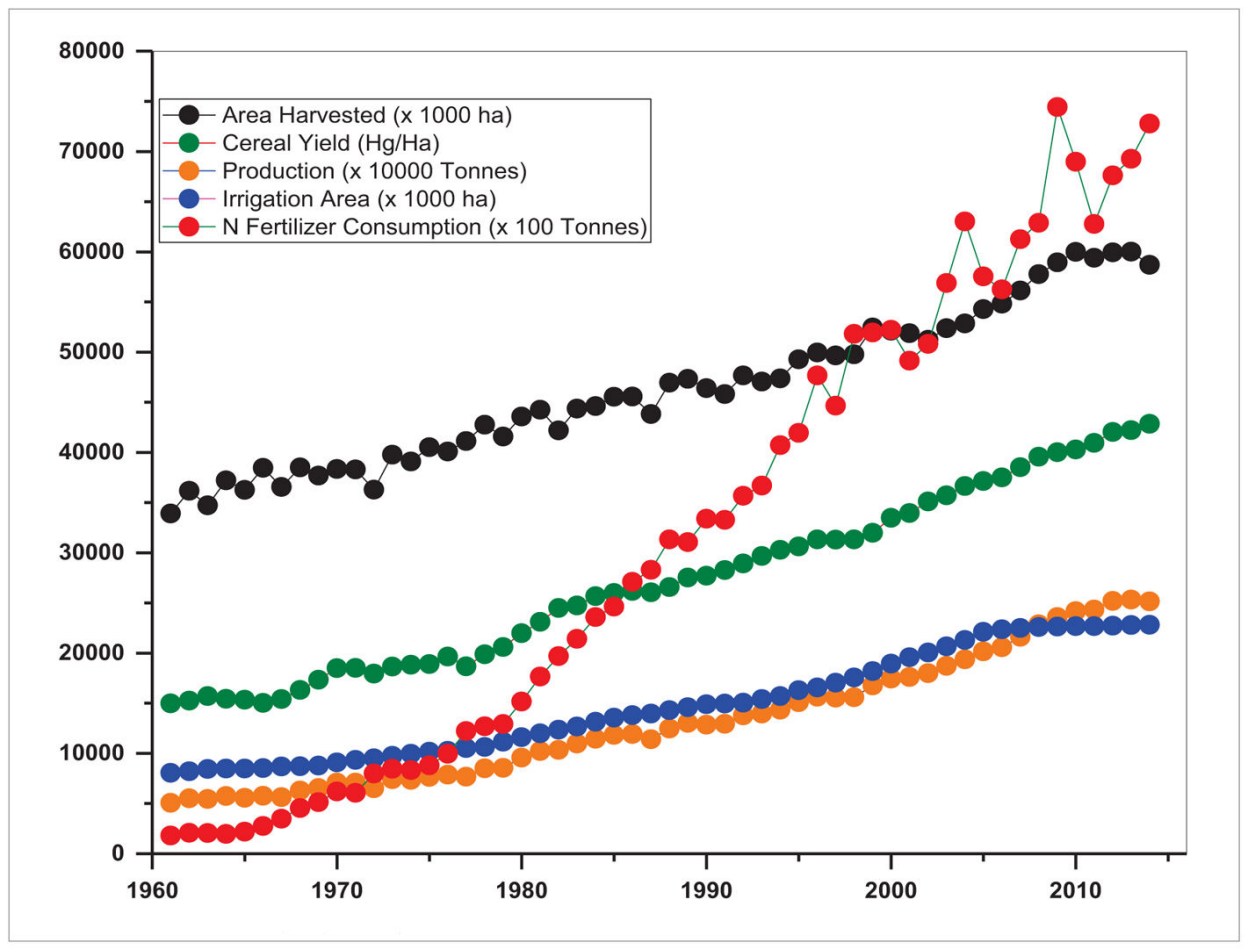
Trends in agricultural intensification indicators in Southeast Asia.

**Figure 16:**
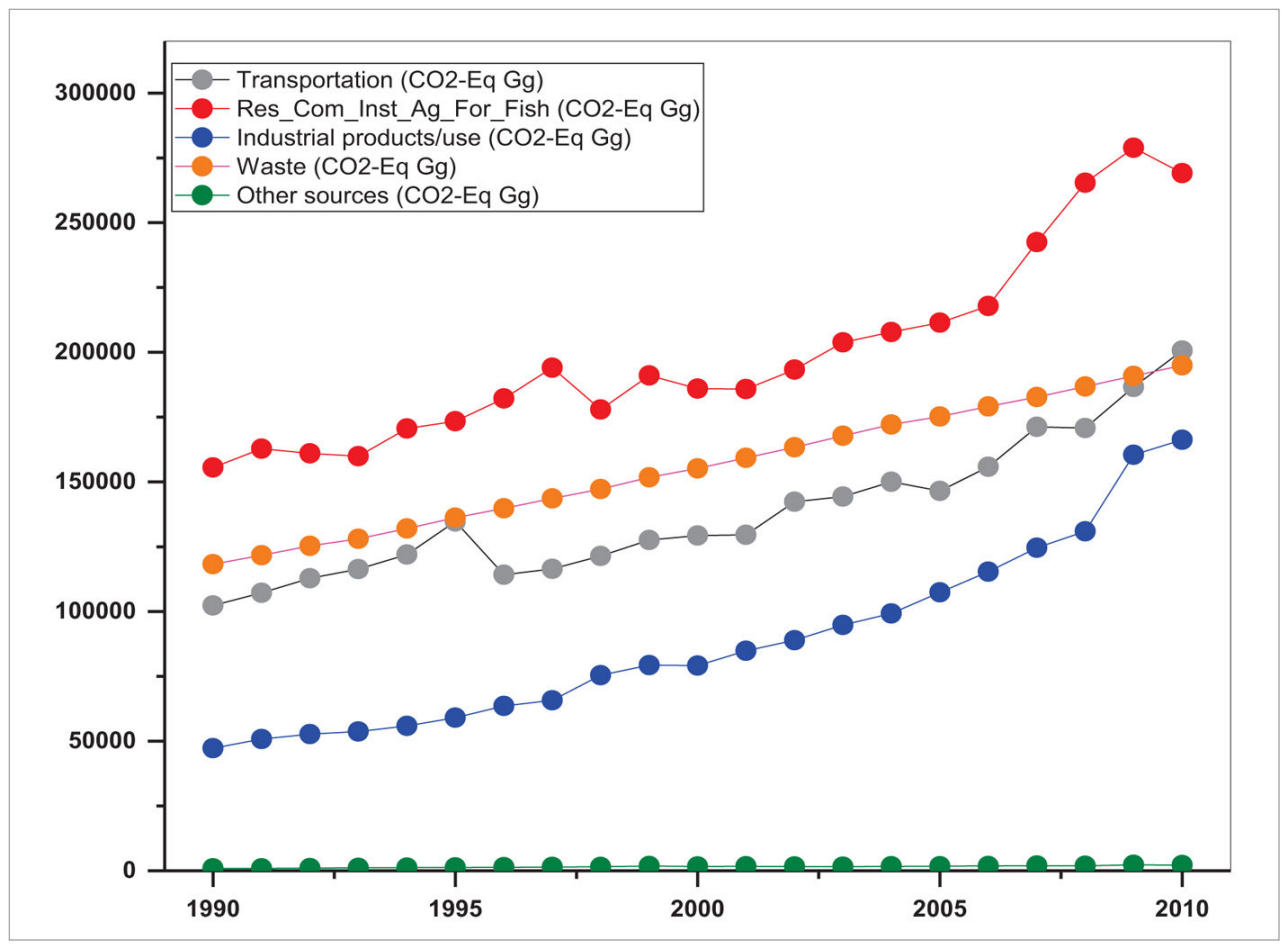
Emissions from different sectors in South Asia (1990–2010).

Our analysis of agricultural datasets from 1960–2015 in SARI countries suggests both agricultural expansion as well as intensification (figures 14–16). The trends suggest that SARI countries have become self-sufficient in agricultural production. However, with a population of about 2.3 billion accounting for 30% of the world’s population, sustaining the agricultural production in these countries without degrading their land resources and environment is a challenging task (Bilsborrow and Carr 2001, Prasad *et al* 2003). Trends in area harvested, food production, cereal yield, nitrogenous fertilizer consumption, and irrigated areas from 1960–2014 are shown in figures 14–15. From these figures, one major difference can be observed in South Asia, i.e. stagnation in terms of harvested agricultural area (figure 14) as compared to Southeast Asia where it is still increasing (figure 15). An increase in production has to be achieved within a limited cropped area which happened through intensified agriculture, as evident in nitrogen fertilizer consumption which was relatively high in South Asia compared to Southeast Asia. For example, from 1960–2014, in South Asia, the area harvested increased by a factor of 1.19, the production by 3.53, cereal yield by 2.96, irrigated areas by 2.4 and nitrogen fertilizer consumption by 61.69. In contrast, in Southeast Asia, the area harvested increased by a factor of 1.73, the production by 4.95, cereal yield by 2.86, irrigated areas by 2.83 and the nitrogen fertilizer consumption by 40.09. These aggregated results may not be sufficient to explain just how agricultural expansion and intensification interacted with subsistence goals, economic development and natural resource management under various circumstances. However, it is well known that agricultural intensification through nitrogen application or irrigation needs careful management, to avoid soil degradation or negative water quality impacts. We infer that understanding the linkages and interdependence among the multiple goals of agricultural production, economic development and environment stewardship is one of the priority areas of agricultural land use research in SARI countries and requires an integrated approach. We also realize an increasing need to develop consistent regional LCLUC products and information with improved spatial and temporal resolutions to be used by SARI scientific communities as well as resource managers to address the above issues and for development planning and policy-making.

## 4. Development of high resolution spatially disaggregated emission inventories are needed to aid in pollution mitigation

**Figure 17:**
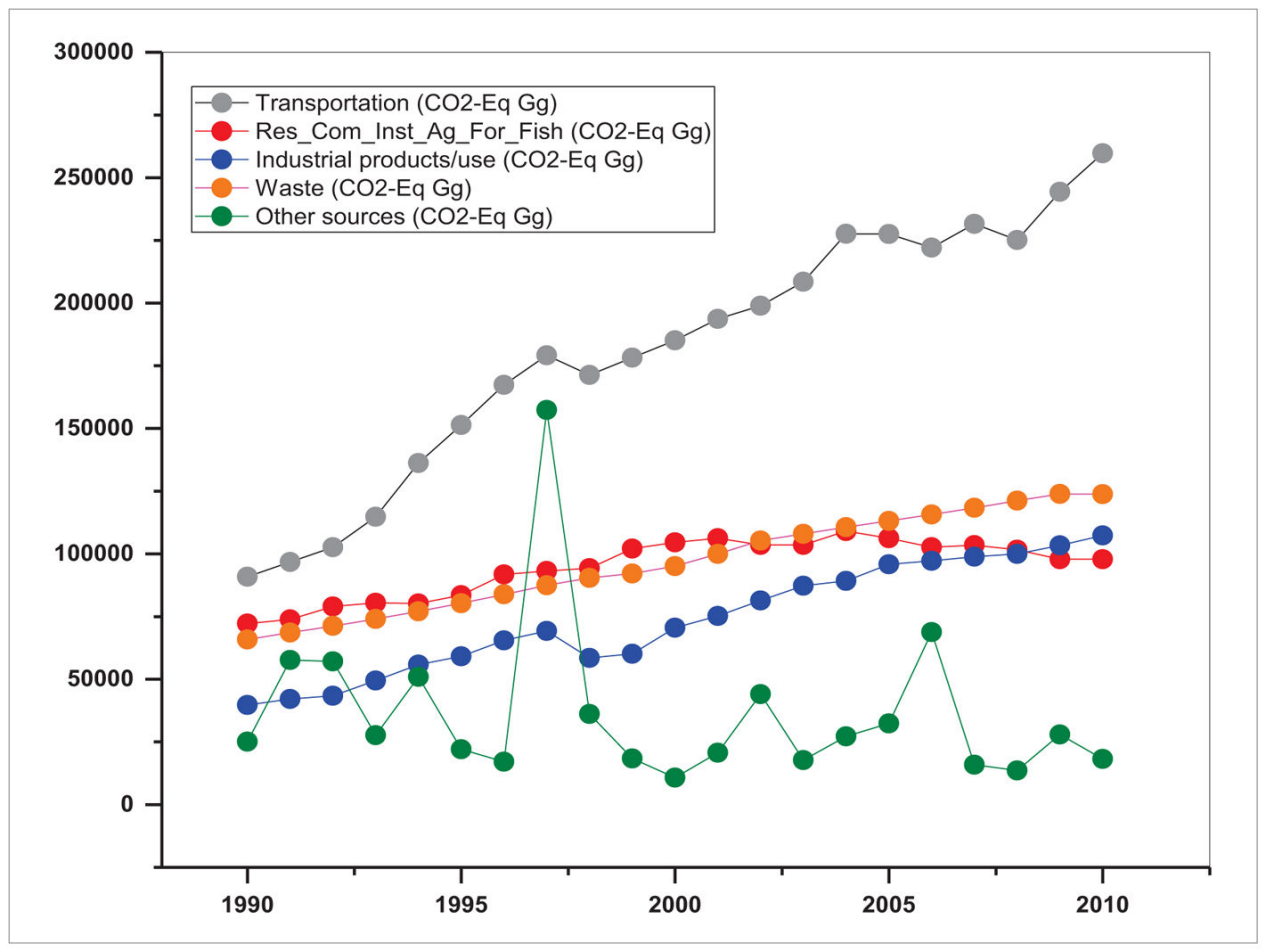
Emissions from different sectors in Southeast Asia (1990–2010).

Land use practices also impact air quality by contributing to GHG emissions and affecting aerosol composition (Fargione *et al* 2008, Popp *et al* 2017). The emissions of GHGs depend on the development pathways governed by land use, agriculture, technological and energy drivers (Shukla *et al* 2003). Integrating land use change science with the drivers and emissions is an important research area as the resulting information can drive policy interventions (Wise *et al* 2009, Riahi *et al* 2017). Specifically, development of emission inventories disaggregated at multiple levels such as by geography, land use type, economic sector, technologies, fuels, etc, at multiple spatial scales is of prime significance. Trends in emissions in terms of CO_2_-equivalent (Gg) for S/SEA countries from 1990–2010 are given in figures 16 and 17. These results suggest sectoral differences in emission inventories and trends. For example, in South Asia, the residential sector followed by transportation and the waste sector are three major sources of GHGs whereas in Southeast Asia, transportation followed by waste and industrial products are major sources of GHGs. Over a period of 20 years in South Asia, transportation emissions increased by a factor of 1.43, residential/commercial/institutional and agriculture/forestry/fishing (AFF) by 1.93, industrial products by 3.54, waste sector by 1.65 and other emissions by 1.51. In contrast in Southeast Asia, the transportation emissions increased by a factor of 2.85, residential/commercial/institutional and AFF by 1.35, industrial products use by 2.70, waste sector by 1.87 and other sources by a factor of 0.72. Emission totals and growth for individual countries might vary, however, here we showcase the variation in sectoral emissions. For most of the countries, the source data is aggregated at a country level and mostly constant emission factors are used in the calculations. Thus, to obtain region specific information on activity data and emission factors, it is necessary to collaborate with emission inventory developers in each country. It is also important to characterize emissions from new sources such as in Sahu *et al* (2017), with an emissions inventory from rapidly growing bagasse-based cogeneration technology in Indian sugar mills for the first time. Also, there is a strong need to integrate satellite and ground-based methodologies to develop and refine emission inventories and operational GHG monitoring systems. Such an attempt is underway to account for all anthropogenic emission sources entitled Regional Emission Inventory in Asia (REAS) (Ohara *et al* 2007). REAS is the first inventory to integrate historical, present, and future emissions in Asia on the basis of a consistent methodology. REAS comprises spatially gridded emissions for major sources including combustion, industrial, agriculture, and other sources at 0.25 × 0.25 degrees on a monthly time step with the target species including sulfur dioxide (SO_2_), nitrogen oxides (NO_x_), carbon monoxide, non-methane volatile organic compounds (NMVOC), PM_10_ (particulate matter), PM_2.5_, black carbon, organic carbon, ammonia, and carbon dioxide (CO_2_). Currently, the REAS is being improved by integrating inverse modeling, chemical transport modeling and observation data to complement emissions. In general, emission estimates in SARI countries have large uncertainties (Saikawa *et al* 2017), especially agricultural emissions and evaporative NMVOC emissions, which need improvement. Development of emission inventories in general can be useful for emissions mitigation and to identify priority sectors and regions for air pollution control.

## 5. Quantifying sources, drivers and impacts of biomass burning in SARI countries is an urgent priority

**Figure 18:**
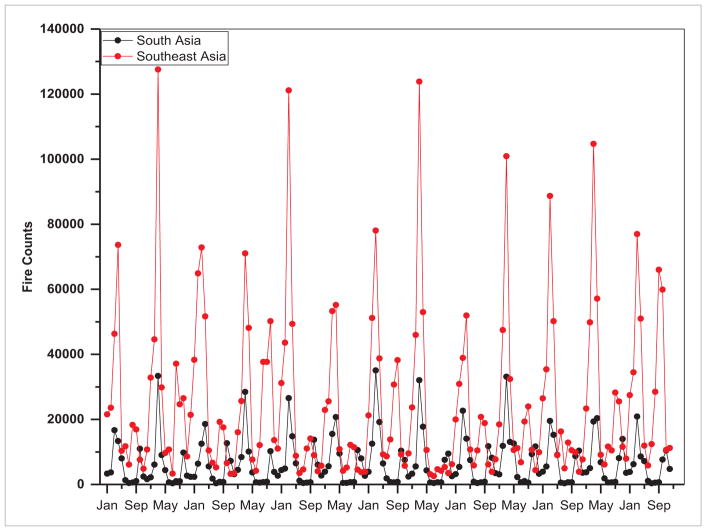
Trends in fire counts retrieved from MODIS (2003–2015) (Aqua and Terra combined data).

The majority of the rural population in SARI countries still depend on natural resources from the local environment for subsistence living. For example, in northeast India, Myanmar, Indonesia, Laos, northern Thailand and Cambodia, deforestation due to slash and burn agriculture is prevalent which has a significant impact on air pollution from biomass burning. Also, burning of agricultural residues for land clearing purposes is pervasive in India, Pakistan, Myanmar, Vietnam, Thailand, Laos and Cambodia. Remote sensing can help to answer vegetation-fire related questions such as amount of fuel availability, fire start and end periods, area burned, and the amount of biomass burned (Chuvieco *et al* 2008). Fire monitoring from satellites began in the 1990s developing algorithms and global datasets at 1 km (~0.6 mi) resolution using data from the Advanced Very High Resolution Radiometer (AVHRR) (Robinson 1991, Kaufman *et al* 1998). Since then, major advances in fire monitoring have been achieved by using data from MODIS (Justice *et al* 2002, Roy *et al* 2008, Giglio *et al* 2003) and Landsat (Smith *et al* 2007). Trends in fire counts as detected by MODIS (Aqua and Terra) satellite for S/SEA from 2003–2015 are given in figure 16. On average, fires in Southeast Asia are higher by a factor of 3.74 compared to South Asia (figure 18). In addition to MODIS, currently operational, data from the Suomi National Polar-orbiting Partnership (NPP) Visible Infrared Imager Radiometer Suite (VIIRS) are providing another step forward in space-based fire monitoring capability. In particular, the 375 m VIIRS fire product (VNP14IMG) can detect small fires such as in agricultural landscapes due to a higher spatial resolution than MODIS (Schroeder *et al* 2014). Landsat data with 30 m resolution has a high potential to detect fires (Kumar and Roy 2017). A paper by Elvidge *et al* (2015), successfully demonstrates the potential of short-wave infrared (SWIR) and long-wave infrared (LWIR) nighttime Landsat data for detecting fires over peatlands in Sumatra and Kalimantan, Indonesia. In addition, future instruments onboard the Joint Polar Satellite System (JPSS), the Geostationary Operational Environmental Satellite (GOES)-R, and the European Space Agency’s Sentinels have high potential to improve the satellite fire retrievals.

An important need with respect to satellite remote sensing of fires is an accuracy assessment of derived products. In SARI countries, there are very few studies which have documented the accuracy of satellite fire products (Tanpipat *et al* 2009). In addition, although the drivers of vegetation fires are mostly anthropogenic, in several SARI countries, climate also impacts fires. For example, vegetation and peatland fires in Southeast Asia have been attributed to a combination of El Niño-induced droughts and anthropogenic land-use changes (Langner and Siegert 2009, Gaveau *et al* 2014). Addressing the interrelationships between climate variables and fires is of high relevance to SARI countries. Further, several studies have shown that aerosols and pollutants from biomass burning can exceed standard levels (Badarinath *et al* 2009, Ikeda and Tanimoto 2015, Sugimoto *et al* 2015) and Vadrevu *et al* 2015) and can be transported long distances and persist for weeks to months, impacting not only air quality, but also biogeochemical cycles, smoke concentrations Marlier *et al* (2015), atmospheric chemistry (Vadrevu *et al* 2013, Tanimoto *et al* 2015), weather, and climate (Seinfeld *et al* 2004, Cristofanelli *et al* 2014) including ozone concentrations (Sonkaew and Macatangay 2015). In addition, biomass burning pollutants can have significant health impacts causing increased respiratory ailments, eye irritation leading to increased medication use and exacerbated asthma (Sastry 2002, Laumbach and Kipen 2012). It is therefore important to more accurately characterize the sources, drivers and impacts of biomass burning in SARI countries.

## 6. Long term GHG and aerosol monitoring networks are needed to benefit scientific understanding and to inform the policy community

In SARI countries, there are very few ground-based GHG and aerosol monitoring networks. Monitoring networks with ground-based instrumentation can provide routine observations needed to assess the impact of the different atmospheric parameters on the local climate (Kant *et al* 2000, Iriana *et al* 2016, Nguyen *et al* 2015 and Ou-Yang *et al* 2015). Regular monitoring of GHGs and aerosols can have large benefits in terms of pollution quantification and mitigation (Prasad *et al* 2000, Badarinath *et al* 2009). In addition, monitoring networks can also serve as a platform for international collaborations to enhance research activities. For example, one of the popular ground-based instrumentation networks for measuring aerosols at a global scale is the Aerosol Robotic Network (AERONET). AERONET is a federated instrument network of ground-based Sun photometers that derive aerosol optical depth (AOD) at a number of visible and near-infrared wavelengths from direct observations of the Sun (Holben *et al* 1998). AERONET provides continuous cloud-screened observations of spectral AOD, precipitable water, and inversion aerosol products in diverse aerosol regimes. Inversion products are retrieved from almucantar scans of radiance as a function of scattering angle and include products such as aerosol volume size distribution, aerosol complex refractive index, optical absorption (single scattering albedo) and the aerosol scattering phase function. AERONET data have been critical to the success of satellite aerosol missions like MISR and MODIS to help retrieve aerosol information from satellites. Algorithm performance is typically evaluated by comparing satellite retrievals with the ground-based observations, which are taken to represent the truth. Discrimination of aerosol types—such as mineral dust, smoke, and anthropogenic pollutants from satellite data is possible through validation from ground-based measurement networks such as AERONET.

Another ground-based network is SKYNET (http://atmos2.cr.chiba-u.jp/skynet/). SKYNET observes optical and microphysical properties of aerosols and clouds and atmospheric radiation at different locations. The SKYNET monitoring sites are equipped with one or more sky looking radiometers. To strengthen the ability of SKYNET, simultaneous measurements with other instruments such as a pyranometer, pyrgeometer, microwave radiometer, absorption meter, cloud camera, Lidar (Sugimoto *et al* (2015), and MAX-DOAS are also included for some selected sites. SKYNET instrumentation helps the quantitative evaluation of long-term variations of aerosols, clouds, and atmospheric radiation, to understand their effects on climate through aerosol–cloud–radiation interactions. In addition, the data are highly useful for validation of satellite observations (e.g. GOSAT, GOSAT-2, GCOM-C, EarthCARE, and Himawari-8) including climate model simulations and data assimilation activities (Sano *et al* 2003, Tanré *et al* 2011, Kosmopoulos *et al* 2017). These aerosol networks also provide important inputs for atmospheric correction of satellite data.

In order to meaningfully compare different satellite-based reflectance products, it will be necessary to better understand several data characteristics, including spatial and spectral differences including directional effects as influenced by aerosols—for which ground-based networks provide important inputs. In addition to robust radiative transfer codes, ground-based instrumentation is a must for generating accurate atmospherically corrected products useful for Earth observation studies (Vermote *et al* 2002).

In contrast to aerosol measuring networks, there are very few networks focusing on measuring GHGs (CO_2_, CH_4_ and NO_x_). Measurements of GHGs on a long-term basis can help to address public health issues, quantify trends and contribute to climate change studies, thus, GHG monitoring networks are very much needed.

## 7. Building air quality modeling research community in SARI countries is a priority

In addition to observational capabilities—gaps and inconsistencies remain with respect to emissions research in SARI countries. Models are necessary to address the wide range of questions regarding emissions, their transformation, transport and deposition at a variety of scales. For example, urban areas in several SARI countries are highly polluted and also impacted by complex weather phenomena (Talukdar *et al* 2017). Thus, atmospheric models can help to understand the coupling between pollutants, weather, chemical, and radiative processes in the atmosphere, such as the Weather Research Forecasting-Chemistry (WRF-Chem) and the Community Multiscale Air Quality (CMAQ) models. In general, the study of air quality problems requires a multi-disciplinary approach and models can assist in providing key predictive capabilities. In addition to process based and empirical models, it is also important to develop models that characterize the exposure of people and ecosystems to pollutants and health risks. Measurements are one way of gathering this information, but models can assist in air pollution health risk assessments (Anenberg *et al* 2016) in data sparse regions. The results from these health risk models can be used to communicate the impact of pollutants or changes in air pollution in different socio-economic, environmental and policy circumstances. For example, using the Asia-Pacific Integrated Assessment End-use model, Mittal *et al* (2015) and Xing *et al* (2015) focus on generating alternate scenarios for air pollution mitigation from the road transportation sector in India. In several SARI countries, the modeling community is weak and there is a need to train researchers and guide them towards the cutting-edge research in the fields of Earth and atmospheric science modeling. Higher education programs in SARI countries should cover modeling aspects by strengthening the domain knowledge, computer coding, and transferable research skills. Training researchers with real-world examples through hands-on practical sessions and facilitating participants to work with their own data, and use of open-source software can yield useful results. Finally, we advocate more funding from both government, and private sector for building modeling research communities to address air pollution issues in the region.

## 8. Development of sustainable technologies for pollution mitigation through research and development is very much needed

Comparative analysis revealed that strategies targeted at the mitigation of local pollution like PM, SO_2_ and NO_x_ also shows greater potential in reducing CO_2_ emissions (as a non-target emission). In general, strategies to mitigate CO_2_ emissions may be based on technological change, economic incentives, and institutional frameworks. They range from using the carbon sequestering potential of afforestation to demand-side or supply-side oriented measures in the energy sector, and even so-called geo- and cosmo-engineering (Riahi and Roehrl 2000). The ‘Special Report on Renewable Energy Sources and Climate Change Mitigation’ by the IPCC highlights multiple renewable energy options including bioenergy, direct solar energy, geothermal energy, hydropower, tidal power and wind energy as alternate sources to curb GHGs (www.ipcc.ch/report/srren/). While these technologies are recognized as important, more focus in terms of both research and development and the associated funding is needed in the Asian countries to test the above options. An integrated analysis of the mitigation potential, costs and benefit analysis should be thoroughly undertaken to arrive at the best management options to reduce GHGs.

## 9. SARI countries require capacity building and training to address environmental problems

Capacity building is typically defined as the development and strengthening of human and institutional resources (United Nations 2006). It is widely acknowledged that capacity building will be successful only through the effective participation of public, private and governmental organizations. In addition, to address scientific questions in a robust manner and to develop practical solutions to environmental problems, communities, organizations and individuals need to acquire a variety of skills and knowledge. SARI meetings in different countries (Myanmar, Vietnam, Thailand, India, and Indonesia) highlighted the need for strong capacity building and training activities in the region. Specific to air pollution mitigation, most of the national programs are understaffed and lack sufficient resources to tackle the problem. Thus, we infer that developing effective infrastructures and programs in conjunction with training and education as highly important to solve environmental and pollution problems. In addition, we also infer the need to foster better communication and cooperation amongst the science community and with the resource management community to address LCLUC related air pollution mitigation in SARI countries.

## References

[R1] Anenberg SC (2016). Survey of ambient air pollution health risk assessment tools. Risk Anal.

[R2] Badarinath KVS (2009). Variations in CO, O_3_ and black carbon aerosol mass concentrations associated with planetary boundary layer (PBL) over tropical urban environment in India. J Atmos Chem.

[R3] Barbier EB, Burgess JC (2001). The economics of tropical deforestation. J Econ Surv.

[R4] Bilsborrow RE, Carr DL, Lee DR, Barrett CB (2001). Population, agricultural land use and the environment in developing countries. Tradeoffs or Synergies? Agricultural Intensification, Economic Development and the Environment.

[R5] Carr DL, Suter L, Barbieri A (2005). Population dynamics and tropical deforestation: state of the debate and conceptual challenges. Popul Environ.

[R6] Chuvieco E, Giglio L, Justice C (2008). Global characterization of fire activity: toward defining fire regimes from Earth observation data. Glob Change Biol.

[R7] Cristofanelli P (2014). Transport of short-lived climate forcers/pollutants (SLCF/P) to the Himalayas during the South Asian summer monsoon onset. Environ Res Lett.

[R8] Elvidge CD (2015). Long-wave infrared identification of smoldering peat fires in Indonesia with nighttime Landsat data. Environ Res Lett.

[R9] Fargione J (2008). Land clearing and the biofuel carbon debt. Science.

[R10] Foell W (1995). Energy use, emissions, and air pollution reduction strategies in Asia. Water Air Soil Poll.

[R11] FAOSTAT (2017). www.fao.org/faostat/en/#home.

[R12] Gaveau DL (2014). Four decades of forest persistence, clearance and logging on Borneo. PLoS ONE.

[R13] Geist HJ, Lambin EF (2002). Proximate causes and underlying driving forces of tropical deforestation: tropical forests are disappearing as the result of many pressures, both local and regional, acting in various combinations in different geographical locations. BioScience.

[R14] Giglio L (2003). An enhanced contextual fire detection algorithm for MODIS. Remote Sens Environ.

[R15] Gurjar BR, Ravindra K, Nagpure AS (2016). Air pollution trends over Indian megacities and their local-to-global implications. Atmos Environ.

[R16] Gustafsson Ö (2009). Brown clouds over South Asia: biomass or fossil fuel combustion?. Science.

[R17] Holben BN (1998). AERONET—A federated instrument network and data archive for aerosol characterization. Remote Sens Environ.

[R18] Iriana W (2016). Measurement of carbon dioxide flux from tropical peatland in Indonesia using the nocturnal temperature-inversion trap method. Environ Res Lett.

[R19] Ikeda K, Tanimoto H (2015). Exceedances of air quality standard level of PM_2.5_ in Japan caused by Siberian wildfires. Environ Res Lett.

[R20] Justice CO, Gutman G, Vadrevu KP (2015). NASA land cover and land use change (LCLUC): an interdisciplinary research program. J Environ Manage.

[R21] Justice CO (2002). The MODIS fire products. Remote Sens Environ.

[R22] Kammen DM, Sunter DA (2016). City-integrated renewable energy for urban sustainability. Science.

[R23] Kumar SS, Roy DP (2017). Global operational land imager Landsat-8 reflectance-based active fire detection algorithm. Int J Digital Earth.

[R24] Kant Y (2000). Studies on aerosol optical depth in biomass burning areas using satellite and ground-based observations. Infrared Phys Techn.

[R25] Kaufman YJ (1998). Potential global fire monitoring from EOS-MODIS. J Geophys Res Atmos.

[R26] Koh LP, Wilcove DS (2008). Is oil palm agriculture really destroying tropical biodiversity. Conserv Lett.

[R27] Koplitz SN (2017). Burden of disease from rising coal-fired power plant emissions in Southeast Asia. Environ Sci Technol.

[R28] Krey V (2012). Urban and rural energy use and carbon dioxide emissions in Asia. Energy Econ.

[R29] Kosmopoulos PG (2017). Dust impact on surface solar irradiance assessed with model simulations, satellite observations and ground-based measurements. Atmos Meas Tech.

[R30] Kuusela OP, Amacher GS (2016). Changing political regimes and tropical deforestation. Environ Resour Econ.

[R31] Lane JE (2017). ASIA: Economic success but uncertain future. Res Econ Manage.

[R32] Langner A, Siegert F (2009). Spatiotemporal fire occurrence in Borneo over a period of 10 years. Glob Change Biol.

[R33] Laumbach RJ, Kipen HM (2012). Respiratory health effects of air pollution: update on biomass smoke and traffic pollution. J Allergy Clin Immunol.

[R34] Marlier ME (2015). Fire emissions and regional air quality impacts from fires in oil palm, timber and logging concessions in Indonesia. Environ Res Lett.

[R35] Mittal S (2015). Air pollution co-benefits of low carbon policies in road transport: a sub-national assessment for India. Environ Res Lett.

[R36] Nguyen TT (2015). Particulate matter concentration mapping from MODIS satellite data: a Vietnamese case study. Environ Res Lett.

[R37] Ohara T, Murano K (2001). Numerical simulation of the springtime trans-boundary air pollution in East Asia. Water Air Soil Poll.

[R38] Ohara T (2007). An Asian emission inventory of anthropogenic emission sources for the period 1980–2020. Atmos Chem Phys.

[R39] Ou-Yang CF (2015). Impact of equatorial and continental airflow on primary greenhouse gases in the northern South China Sea. Environ Res Lett.

[R40] Popp A (2017). Land-use futures in the shared socio-economic pathways. Glob Environ Change.

[R41] Prasad VK (2003). Trends in food production and nitrous oxide emissions from the agriculture sector in India: environmental implications. Reg Environ Change.

[R42] Prasad VK (2000). NO_x_ emissions from biomass burning of shifting cultivation areas from tropical deciduous forests of India—estimates from ground-based measurements. Atmos Environ.

[R43] Riahi K, Roehrl RA (2000). Greenhouse gas emissions in a dynamics-as-usual scenario of economic and energy development. Technol Forecast Soc Change.

[R44] Riahi K (2017). The shared socioeconomic pathways and their energy, land use, and greenhouse gas emissions implications: an overview. Glob Environ Change.

[R45] Robinson JM (1991). Fire from space: global fire evaluation using infrared remote sensing. Int J Remote Sens.

[R46] Roy DP (2008). Multi-temporal MODIS—Landsat data fusion for relative radiometric normalization, gap filling, and prediction of Landsat data. Remote Sens Environ.

[R47] Sahu SK, Ohara T, Beig G (2017). The role of coal technology in redefining India’s climate change agents and other pollutants. Environ Res Lett.

[R48] Saikawa E (2017). Uncertainties in emissions estimates of greenhouse gases and air pollutants in India and their impacts on regional air quality. Environ Res Lett.

[R49] Sano I (2003). Calibration and validation of retrieved aerosol properties based on AERONET and SKYNET. Adv Space Res.

[R50] Sastry N (2002). Forest fires, air pollution, and mortality in Southeast Asia. Demography.

[R51] Seinfeld JH (2004). ACE-ASIA: regional climatic and atmospheric chemical effects of Asian dust and pollution. Bull Am Meteorol Soc.

[R52] Schroeder W (2014). The New VIIRS 375 m active fire detection data product: algorithm description and initial assessment. Remote Sens Environ.

[R53] Shandra JM, Rademacher H, Coburn C (2016). The World Bank and organized hypocrisy? A cross-national analysis of structural adjustment and forest loss. Environ Sociol.

[R54] Shukla PR, Garg A, Kapshe M, Shukla PR, Sharma SK, Ravindranath NH, Garg A, Bhattacharya S (2003). Climate Change and India: Vulnerability Assessment and Adaptation.

[R55] Smith AMS (2007). Production of Landsat ETM+ reference imagery of burned areas within Southern African savannahs: comparison of methods and application to MODIS. Int J Remote Sens.

[R56] Sovacool BK (2007). Solving the oil independence problem: is it possible?. Energy Policy.

[R57] Sonkaew T, Macatangay R (2015). Determining relationships and mechanisms between tropospheric ozone column concentrations and tropical biomass burning in Thailand and its surrounding regions. Environ Res Lett.

[R58] Sugimoto N (2015). Aerosol characteristics in Phimai, Thailand determined by continuous observation with a polarization sensitive Mie–Raman lidar and a sky radiometer. Environ Res Lett.

[R59] Tanimoto H (2015). Interannual variability of nitrogen oxides emissions from boreal fires in Siberia and Alaska during 1996–2011 as observed from space. Environ Res Lett.

[R60] Tanpipat V, Honda K, Nuchaiya P (2009). MODIS hotspot validation over Thailand. Remote Sensing.

[R61] Tanré D (2011). Remote sensing of aerosols by using polarized, directional and spectral measurements within the A-Train: the PARASOL mission. Atmos Meas Tech.

[R62] Talukdar S, Soumyajyoti J, Maitra A (2017). Dominance of pollutant aerosols over an urban region and its impact on boundary layer temperature profile. J Geophys Res Atmos.

[R63] United Nations (2006). Definition of basic concepts and terminologies in governance and public administration Note.

[R64] Vadrevu KP, Giglio L, Justice C (2013). Satellite based analysis of fire–carbon monoxide relationships from forest and agricultural residue burning 2003–2011. Atmos Environ.

[R65] Vadrevu KP (2015). Vegetation fires, absorbing aerosols and smoke plume characteristics in diverse biomass burning regions of Asia. Environ Res Lett.

[R66] Vermote EF, Saleous ENZ, Justice CO (2002). Atmospheric correction of MODIS data in the visible to middle infrared: first results. Remote Sens Environ.

[R67] Vijay V (2016). The impacts of oil palm on recent deforestation and biodiversity loss. PloS ONE.

[R68] Wise M (2009). Implications of limiting CO_2_ concentrations for land use and energy. Science.

[R69] Wilcove DS, Koh LP (2010). Addressing the threats to biodiversity from oil-palm agriculture. Biodivers Conserv.

[R70] Xing R (2015). An impact assessment of sustainable technologies for the Chinese urban residential sector at provincial level. Environ Res Lett.

[R71] Ziegler AD, Fox JM, Xu J (2009). The rubber juggernaut. Science.

